# Book Review

**DOI:** 10.1120/jacmp.v6i2.2133

**Published:** 2005-05-21

**Authors:** Amir Huda

**Affiliations:** ^1^ Department of Physics California State University 2345 E. San Ramon Avenue, M/S MH37 Fresno California 93740‐8031

## Abstract

*Primer on MR Imaging of the Abdomen and Pelvis*, by Diego R. Martin, Michèle A. Brown, and Richard C. Semelka, John Wiley & Sons, Inc. 2005; ISBN 0‐471‐37340‐0; list price $59.95 (paperback)

*Webster's* dictionary defines a primer as “a book covering the basic elements of a subject.” This book fulfills that definition not with respect to the fundamentals of MR imaging but how MR imaging can be applied for diagnostic evaluation and management of diseases of the abdomen and the pelvis. The authors' primary motivation for the book stems from the observation of “a gap between the utility and the utilization of MRI.” As such, its primary audience is the clinician, that is, radiology resident, fellow, or practicing radiologist. This book may serve as a recipe for the clinician to learn how differences of T2, T1, and proton density in tissue, blood, and fluids can be exploited, along with administration of contrast and suppression techniques in a large variety of sequences for a myriad of disorders and diseases to obtain the best possible disease conspicuity for differential diagnosis. In addition, the sequences are also weighed against examination time, a primary and important factor governing many procedures in the clinic.

While the maze of possibilities may seem overwhelming for some who are accustomed to obtaining a simple cross‐sectional set of images in other modalities such as CT, it is precisely these possibilities that promise a wealth of detail and features with greater contrast resolution to the radiologist that are unobtainable in other ways. Thus, a resource such as this may prove quite valuable to the practicing radiologist.

After an introductory chapter, the book is sectioned into chapters according to the organ system, that is, liver, gallbladder and bile ducts, pancreas, spleen, kidneys, adrenal glands, gastrointestinal tract, retro peritoneum, peritoneum, bladder, male pelvis, and female pelvis. The format of each chapter is fairly similar: Each chapter starts out with text and images of the normal organ as seen under different MR sequences. This is followed by sections on particular diseases, along with a large set of images for each disease under different MR sequences, explaining in detail the advantages and disadvantages of a particular sequence. Covering a span of close to 500 pages, using tables, text, images, and special notes guiding the radiologist on the effects of things such as stent placement on the diagnostic sensitivity of the study, the authors outline change in intrinsic properties such as T1 and T2, along with early and late effects of gadolinium administration for different lesions such as cysts, hemangioma, metastases, hamartoma, etc., at different stages; this provides a wealth of information for pattern recognition to aid in diagnosis and management of diseases.

For the physicist, aside from developing an appreciation of why certain sequences are used for certain suspects in the diagnosis and management of diseases, I would have reservations in advocating that this book be a necessary part of the medical physicist's library. The targeted audience is the clinician, and it reaches its audience quite well by providing an armament of tools available for practice in the clinic.

